# Gastroesophageal reflux disease and risk of incident lung cancer: A large prospective cohort study in UK Biobank

**DOI:** 10.1371/journal.pone.0311758

**Published:** 2024-11-11

**Authors:** Ye Liao, Yunfeng Zhou, Xiaorui Zhou, Jing Chen, Zhenhua Chen, Juan Liao, Lu Long

**Affiliations:** 1 Department of Epidemiology and Biostatistics, West China School of Public Health and West China Fourth Hospital, Sichuan University, Chengdu, People’s Republic of China; 2 Departments of Thoracic Surgery, West China School of Public Health and West China Fourth Hospital, Sichuan University, Chengdu, People’s Republic of China; 3 Department of Local Disease Control and Prevention, Sichuan Provincial Center for Disease Control and Prevention, Chengdu, People’s Republic of China; 4 Department of Microbiology Laboratory, Chengdu Municipal Center for Disease Control and Prevention, Chengdu, People’s Republic of China; 5 Department of Gastroenterology, West China School of Public Health and West China Fourth Hospital, Sichuan University, Chengdu, People’s Republic of China; Kyung Hee University School of Medicine, REPUBLIC OF KOREA

## Abstract

**Background:**

Some pathogenic mechanisms suggest a potential relationship between gastroesophageal reflux disease (GERD) and respiratory diseases. However, evidence regarding the association between GERD and lung cancer is mixed. We aim to explore this relationship based on data from the large-scale UK Biobank study.

**Materials and methods:**

We performed a cross-sectional and prospective cohort study in 501,569 (45.58% male) individuals included in the UK Biobank at baseline (2006–2010). The Cox proportional hazards model and logistic regression models were used to assess the relationship between GERD and lung cancer, small cell lung cancer (SCLC), lung squamous cell carcinoma (LUSC), and lung adenocarcinoma (LUAD).

**Results:**

During a mean follow-up of 11.54 years, 3,863 (0.84%) incident lung cancer cases were identified. In the cross-sectional analysis using logistic models, significant associations were found between GERD and prevalent lung cancer cases (**odds ratio [OR] = 1.87, 95% confidence interval [95% CI]: 1.45–2.38**) and subtypes of lung cancer, with an OR (95% CI) of **3.19 (1.47–6.79)** for SCLC, **2.08 (1.33–3.21)** for LUSC, **1.85 (1.15–2.91)** for LUAD. In the follow-up analysis using Cox models, GERD was associated with an increased risk of lung cancer (hazard ratio [**HR] = 1.24, 95%CI: 1.14–1.34**). Similar associations were also observed between GERD and SCLC (**HR = 1.39, 95% CI: 1.09–1.78),** LUSC **(HR = 1.40, 95% CI: 1.18–1.65),** and LUAD **(HR = 1.17, 95% CI: 1.02–1.33**). The risk of lung cancer resulting from GERD was mainly elevated in former smokers (**HR = 1.38, 95% CI: 1.23–1.54)** and current smokers **(HR = 1.18, 95% CI: 1.04–1.34**), but not in never-smokers (HR = 0.89, 95% CI: 0.70–1.14). No significant association was observed in former smokers who had quit smoking for at least 25 years.

**Conclusions:**

We found that GERD was positively associated with an increased risk of lung cancer, especially among smokers. Awareness of this association may be beneficial for prevention and treatment strategies of both diseases.

## 1. Introduction

Lung cancer is the leading cause of both cancer deaths and disease burden globally. In 2022, nearly 2.5 million individuals were diagnosed with lung cancer, in addition to 1.8 million related deaths worldwide [1]. Approximately 30% of lung cancer cannot be attributable to known risk factors such as smoking, high air pollution, and unhealthy lifestyle [2]. It’s crucial to identify potential novel modifiable risk factors for preventing its development.

Gastroesophageal reflux disease (GERD) is a chronic digestive disease primarily characterized by frequent regurgitation of stomach acid and bile [3]. Refluxed gastric contents are thought to promote epithelial damage, not only in the esophagus but also in the airway epithelium [4]. Prolonged exposure of the airway epithelium to gastric acid and bile salts may lead to increased oxidative stress, activation of inflammatory mediators and DNA damage that promote carcinogenesis [5, 6]. Therefore, in addition to the known associations between GERD and cancer of the esophagus, larynx and pharynx [7–9], GERD has been suggested to be associated with lung cancer as well. However, the results from the available literature are mixed [10, 11]. Only Mendelian Randomization (MR) study have provided causal evidence of an association between GERD and lung cancer risk in the UK Biobank [12, 13]. We conducted observational studies to supplement these findings and further investigate this epidemiological relationship, offering a different perspective by specifically considering the roles of smoking and smoking cessation.

To further examine the potential associations between GERD and lung cancer, we conducted observational analyses using data from the large, independent, prospective UK Biobank study and also explored the associations with respect to smoking and quitting status.

## 2. Materials and methods

### 2.1 Study design and population

The data for this study came from the UK Biobank, whose design and study population have been described previously [14]. In brief, UK Biobank is a prospective study involving over 500,000 individuals aged 40–69 recruited between 2006 and 2010, with 22 assessment centers across England, Wales, and Scotland (https://www.ukbiobank.ac.uk/). Two separate sub-studies were constructed: a cross-sectional study with lung cancer patients at baseline and participants without lung cancer, and a prospective cohort with participants without lung cancer at baseline to assess the prospective associations between baseline GERD and the incidence of lung cancer during the follow-up among participants free of cancer at baseline. **S1 Fig** presents the participant selection process. All participants provided written informed consent via touchscreen, and the UK Biobank study was granted ethics approval from the North West Multicenter Research Ethical Committee.

### 2.2 Assessment of GERD

GERD at baseline was defined using self-report, International Classification of Diseases (ICD) 10/9 diagnosis, operative procedures linked to hospital inpatient records, and self-reported GERD-related medication use [15]. The presence of any of the mentioned data fields (including medications for GERD) was considered an indication of GERD. Individuals with no entries in the mentioned data fields were considered controls. A detailed description of the GERD definition is provided in **S1 Table**.

### 2.3 Assessment of lung cancer

Data on lung cancer was obtained through linkage to cancer registries. Lung cancer was defined by ICD-10 codes C33-C34. In addition, we considered three major histological subtypes of lung cancer using the International Classification of Diseases for Oncology, third edition code; small cell lung cancer (SCLC): 8041, 8043, 8044, 8045; lung squamous cell carcinoma (LUSC): 8070, 8071, 8072, 8083; lung adenocarcinoma (LUAD): 8140, 8144, 8230, 8250, 8253, 8254, 8256, 8257, 8260, 8265, 8333, 8480, 8551 [16, 17].

### 2.4 Assessment of covariates

Self-reported sociodemographic and lifestyle characteristics included sex (male, female), age at recruitment (years), race (white, non-white), smoking status (never-smokers, former smokers, current smokers), frequency of alcohol intake (daily or almost daily, 3–4 times a week, 1–2 times a week, occasionally, never), and family history of cancer (yes, no, or missing). The Townsend deprivation index was used as a marker of area-based socioeconomic status and analyzed as a continuous variable. Physical activity (low, moderate, high, or missing) was assessed using an adapted version of the International Physical Activity Short Form questionnaire [18]. Baseline health status was extracted from self-report or algorithmically defined outcomes, including history of diabetes (yes, no), hypertension (yes, no), and chronic obstructive pulmonary disease (COPD) (yes, no). Body mass index (BMI) was calculated as weight in kilograms divided by height in meters squared and categorized according to the WHO’s classification (<18.5, 18.5-<25, 25.0-<30.0, ≥30.0 kg/m^2^) [19].

### 2.5 Missing data

The missing rate of covariates ranged from 0 to 19.86% in the cross-sectional study and from 0 to 19.71% in the prospective study (**S2 and S3 Tables**). The study population’s missing values were replaced with the sex-specific median and mode values for continuous and categorical variables, respectively. Due to the rate of missing data for physical activity level and family history of cancer being more than 3%, we created a group named “missing” for these variables [20].

### 2.6 Statistical analysis

We assessed lung cancer risk in participants from the enrolment until the time of lung cancer diagnosis, loss to follow-up, death, or the end of follow-up (December 31, 2020 for England, November 30, 2021 for Scotland and December 31, 2016 for Wales), whichever occurred first.

Cox proportional hazard regression models were performed to calculate the hazard ratio (HR) and 95% confidence intervals (CI) assessing the associations of GERD with risk of incident lung cancer. If the HR = 1 then that predictor does not affect survival. If the HR<1, then the predictor is protective (i.e., associated with improved survival), and if the HR>1, then the predictor is associated with increased risk (or decreased survival) [21]. The proportional hazards assumption was tested using Schoenfeld residuals, and no violations were identified. We calculated the cumulative incidence using the R packages “survival” and plotted the curves using “survminer”. The multivariate models were adjusted for sex, age, race, BMI, smoking status, frequency of alcohol intake, family history of cancer, physical activity, Townsend deprivation index, history of diabetes, history of hypertension and history of COPD.

Logistic regression models were used to examine the association between GERD and prevalent lung cancer in the cross-sectional study. Results are presented as odds ratios (OR) with 95% CI. An OR of 1 means that there is no increase or decrease in risk. An OR that is <1 means that exposure to the risk variable reduces the risk of the event. An OR that is >1 means that the risk is increased. The covariate adjustments in the logistic regression models were consistent with those in the Cox proportional hazards regression models.

To test the robustness and potential variations in different subgroups, subgroup analyses of associations between GERD and risk of incident lung cancer were conducted across categories of sex (male, female), age at recruitment (<60, ≥60 years), BMI (<25, ≥25 kg/m^2^), smoking status (never-smokers, former smokers, current smokers), frequency of alcohol intake (non-excessive alcohol intake, excessive alcohol intake), family history of cancer (yes, no), physical activity (low, moderate, high), diabetes (yes, no), hypertension (yes, no), and COPD (yes, no). Notably, non-excessive alcohol intake was defined as alcohol intake of less than 1–2 times a week, and excessive alcohol intake was defined as alcohol intake of at least 1–2 times a week [22]. Binarizing variables helps simplify subgroup analyses and ensures consistency by reducing complexity.

Several accuracy analyses were performed to assess the robustness of the findings: *1)* excluding participants who developed lung cancer within 2 years; *2)* excluding participants with missing covariate data; *3)* using the 5 multiple imputation technique to impute the missing covariates.

All statistical analyses were performed with R software (version 4.3.2). A *P* value < 0.05 (two-sided) was considered statistically significant.

## 3. Result

### 3.1 Association between GERD and lung cancer at baseline

The characteristics of cross-sectional study participants are presented in **S4 Table**. A total of 501,569 participants were included, of whom 321 had a diagnosis of lung cancer at baseline. Participants with GERD were more likely to be older, smokers, to have a lower socioeconomic status, and tended to have diabetes, hypertension, and COPD compared with participants without GERD.

In the cross-sectional study, compared with participants without lung cancer, GERD was significantly associated with prevalent lung cancer (OR = 1.87, 95% CI: 1.45–2.38). When analyzing GERD and subtypes of lung cancer separately, the results remained significant, with an OR (95% CI) of 3.19 (1.47–6.79) for SCLC, 2.08 (1.33–3.21) for LUSC, and 1.85 (1.15–2.91) for LUAD (all *P* < 0.05) (**Table 1**).

**Table 1:** Association between gastroesophageal reflux disease and lung cancer.

	Lung Cancer		SCLC		LUSC		LUAD	
GERD	Cases^a^	OR (95% CI)^*b*^; *P*	Cases^c^	OR (95% CI)^*b*^; *P*	Cases^*d*^	OR (95% CI)^*b*^; *P*	Cases^*e*^	OR (95% CI)^*b*^; *P*
No	220	1 (referent)	17	1 (referent)	60	1 (referent)	62	1 (referent)
Yes	101	1.87(1.45–2.38) <0.001	13	3.19(1.47–6.79) 0.003	34	2.08(1.33–3.21) 0.001	30	1.85(1.15–2.91) 0.009

^*a*^Number of lung cancer cases in each category is listed.

^*b*^The logistic regression models were adjusted by age (continuous), sex (male or female), race (white, non-white), body mass index (underweight (< 18.5), healthy (18.5 to < 25.0), overweight (25.0 to < 30.0), obesity (≥ 30.0)), Townsend deprivation index (continuous), smoking status (never-smokers, former smokers, current smokers), frequency of alcohol intake (never, occasionally, 1–2 times a week, 3–4 times a week, daily, almost daily), history of diabetes (yes or no), history of hypertension (yes or no), history of chronic obstructive pulmonary disease (yes or no), physical activity (low, moderate, high, missing) and family history of cancer (yes, no, missing).

^*c*^Number of SCLC cases in each category is listed.

^*d*^Number of LUSC cases in each category is listed.

^*e*^Number of LUAD cases in each category is listed.

Abbreviations: GERD, gastroesophageal reflux disease; OR, odds ratio; CI, confidence interval; SCLC, small cell lung cancer; LUSC, lung squamous cell carcinoma; LUAD, lung adenocarcinoma.

### 3.2 GERD and risk of incident lung cancer

458,691 participants were included in the prospective study, with a mean (SD) follow-up of 11.54 (1.78) years. **Table 2** illustrates the distribution of baseline characteristics of the prospective study participants. 58,191 (12.69%) participants reported to have GERD. Compared to those without GERD, participants diagnosed with GERD tended to be older, female, and smokers. They were also more likely to have a BMI of 25 kg/m^2^ or greater, lower socioeconomic status (Townsend deprivation index), a higher prevalence of diabetes, hypertension, and COPD, and a family history of cancer. Additionally, they were more likely to report low levels of physical activity and alcohol intake.

**Table 2:** Baseline characteristics of participants according to the status of gastroesophageal reflux disease in the prospective study.

	Gastroesophageal Reflux Disease	
Characteristics	No	Yes
Total number of patients	400,500	58,191
Time from baseline to end of follow-up		
Mean (SD)	11.57(1.73)	11.34(2.08)
Median (IQR)	11.78(10.99,12.46)	11.68(10.87,12.43)
Range	(0.01–14.80)	(0.01–14.80)
Person-y	4,634,248	659,989
Male (%)	187,446 (46.8)	27,151 (46.7)
Age at baseline (median [IQR]-y)	57.0 (49.0, 63.0)	61.0 (54.0, 65.0)
White (%)	377,955 (94.4)	55,206 (94.9)
BMI (%- kg/m^2^)		
<18.5	2,143 (0.5)	185 (0.3)
18.5-<25	136,491 (34.1)	11,622 (20.0)
25.0-<30.0	171,338 (42.8)	25,396 (43.6)
≥30.0	90,528 (22.6)	20,988 (36.1)
Townsend deprivation index (median [IQR])	-2.18 (-3.67, 0.43)	-1.76 (-3.45, 1.31)
Smoking status (%)		
Never-smokers	226,330 (56.5)	27,855 (47.9)
Former smokers	132,667 (33.1)	23,569 (40.5)
Current smokers	41,503 (10.4)	6,767 (11.6)
Frequency of alcohol intake (%)		
Never	29,741 (7.4)	6,968 (12.0)
Occasionally^*a*^	87,779 (21.9)	15,307 (26.3)
1–2 times a week	105,166 (26.3)	14,244 (24.5)
3–4 times a week	95,312 (23.8)	11,079 (19.0)
Daily or almost daily	82,502 (20.6)	10,593 (18.2)
Family history of cancer (%)		
No	235,015 (58.7)	31,545 (54.2)
Yes	119,027 (29.7)	18,126 (31.1)
Missing	46,458 (11.6)	8,520 (14.6)
Physical activity (%)		
Low	58,714 (14.7)	10,592 (18.2)
Moderate	132,259 (33.0)	17,629 (30.3)
High	133,028 (33.2)	16,078 (27.6)
Missing	76,499 (19.1)	13,892 (23.9)
Diabetes (%)	17,527 (4.4)	5,472 (9.4)
Hypertension (%)	97,432 (24.3)	23,139 (39.8)
COPD (%)	5,250 (1.3)	2,410 (4.1)

*Notes*: Values are median (IQR) for continuous variables and frequencies (percentages) for categorical variables.

Abbreviations: BMI, body mass index; COPD, chronic obstructive pulmonary disease; IQR, inter quartile range.

^*a*^ Includes individuals who drink on special occasions only and individuals who drink 1–3 times a month.

**Fig 1:**
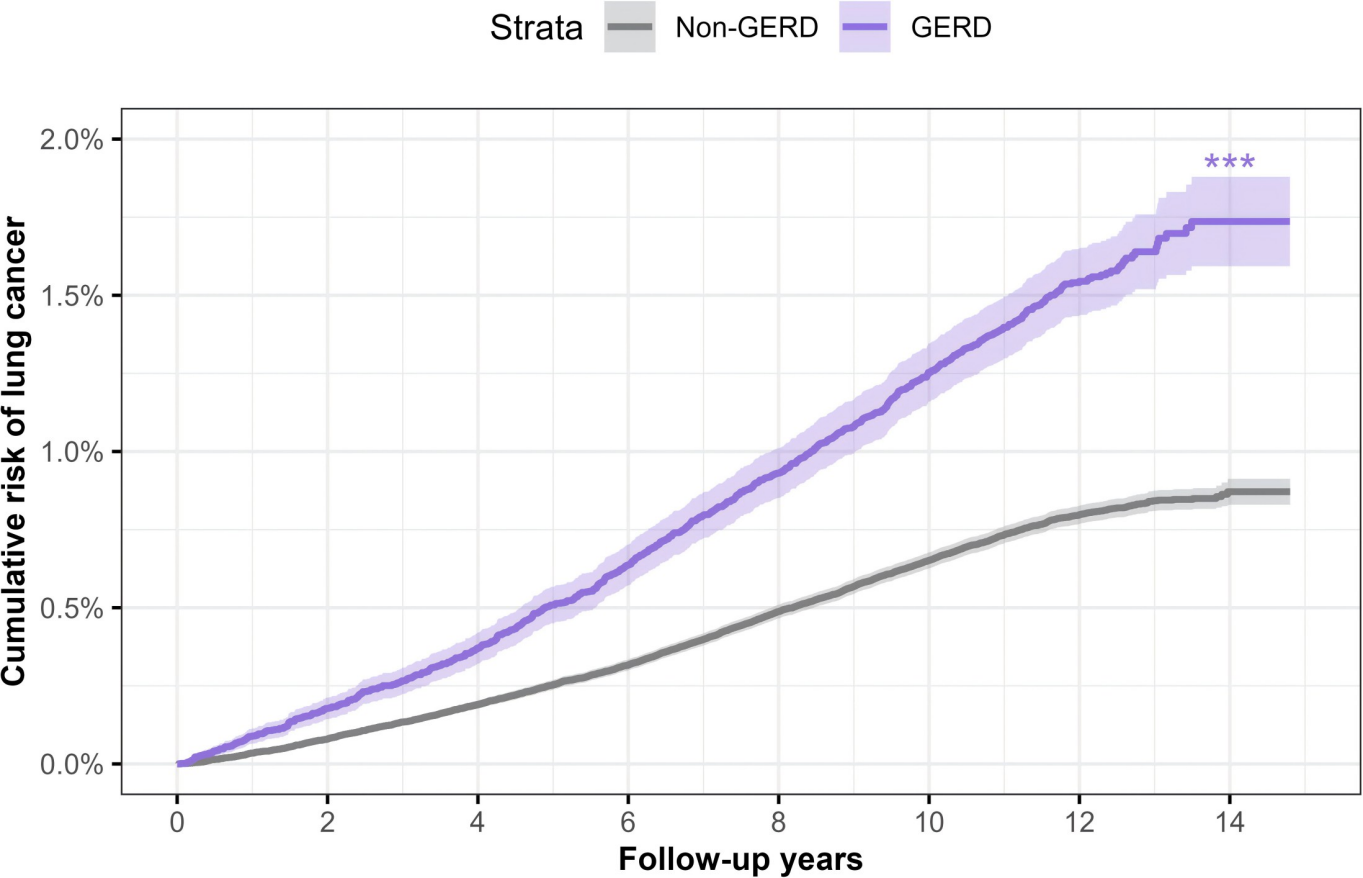
The cumulative risk of lung cancer among individuals with and without gastroesophageal reflux disease. Abbreviations: GERD, gastroesophageal reflux disease. ***p < 0.001.

**Fig 2:**
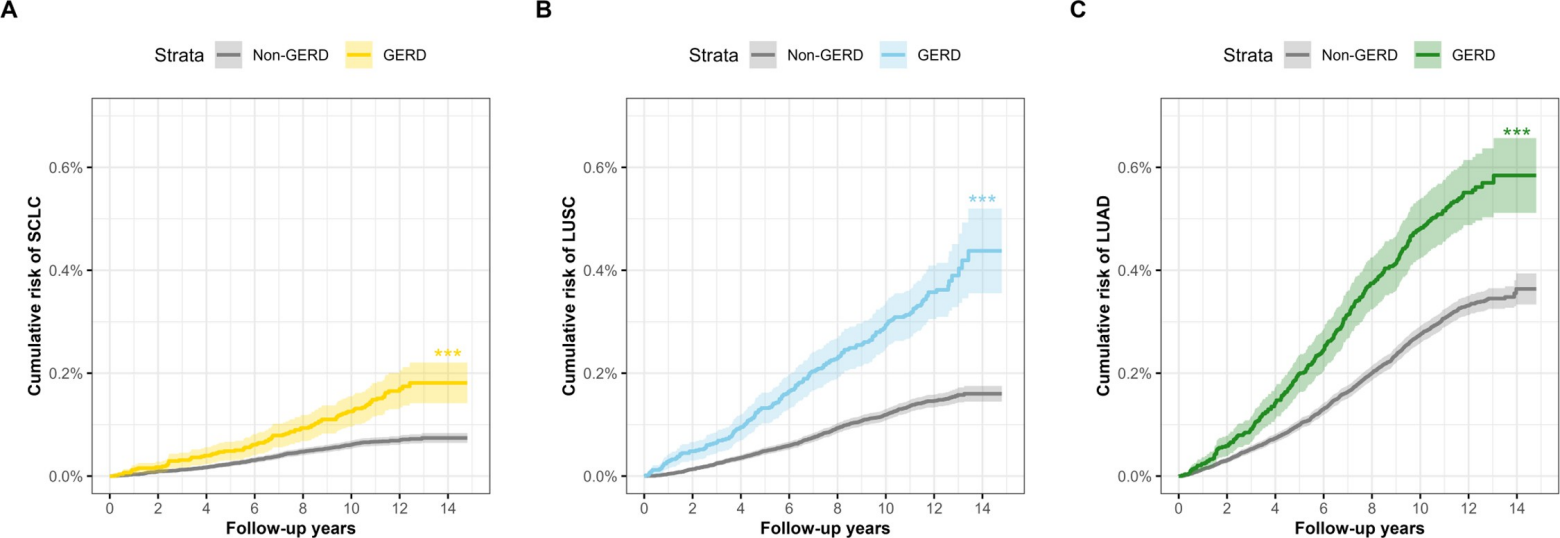
The cumulative risk of histological subtypes of lung cancer among individuals with and without gastroesophageal reflux disease. Abbreviations: GERD, gastroesophageal reflux disease; SCLC, small cell lung cancer; LUSC, lung squamous cell carcinoma; LUAD, lung adenocarcinoma. ***p < 0.001.

During 5,294,236 person-years of follow-up, 3,863 incident lung cancer cases were identified, including 360 SCLC cases, 757 LUSC cases, and 1560 LUAD cases. Compared to participants without GERD, those with GERD typically have a higher cumulative risk of lung cancer. Participants with GERD have a cumulative risk of 1.74% for lung cancer (**Fig 1**), and the cumulative risks for SCLC, LUSC, and LUAD are 0.18%, 0.44%, and 0.58%, respectively (**Fig 2**).

In the prospective study, an association with GERD and incident lung cancer (HR = 1.24, 95% CI: 1.14–1.34, *P* <0.001) was observed in the multivariable Cox proportional hazards regression model (**Table 3**). We further estimated the associations of GERD with three major histological subtypes of lung cancer. The results showed that participants with GERD have an increased risk of incident subtypes of lung cancer, with an HR (95% CI) of 1.39 (1.09–1.78) in SCLC, 1.40 (1.18–1.65) in LUSC, and 1.17 (1.02–1.33) in LUAD, respectively (all *P* <0.05).

**Table 3:** Incident different histologic subtypes of lung cancer in relation to gastroesophageal reflux disease among study participants.

	Lung Cancer		SCLC		LUSC		LUAD	
GERD	Cases^*a*^/ person-years	HR (95% CI)^*b*^; *P*	Cases^*c*^/ person-years	HR (95% CI)^*b*^; *P*	Cases^*d*^/ person-years	HR (95% CI)^*b*^; *P*	Cases^*e*^/ person-years	HR (95% CI)^*b*^; *P*
No	3,028/19,777	1 (referent)	270/1,675	1 (referent)	561/3,652	1 (referent)	1,259/8,296	1 (referent)
Yes	835/5,395	1.24(1.14–1.34) <0.001	90/606	1.39(1.09–1.78) 0.009	196/1,227	1.40(1.18–1.65) <0.001	301/1,847	1.17(1.02–1.33) 0.020

Abbreviations: GERD, gastroesophageal reflux disease; HR, hazard ratio; CI, confidence interval; SCLC, small cell lung cancer; LUSC, lung squamous cell carcinoma; LUAD, lung adenocarcinoma.

^*a*^Number of lung cancer cases in each category is listed.

^*b*^The Cox proportional hazard models were adjusted by age (continuous), sex (male or female), race (white, non-white), body mass index (underweight (< 18.5), healthy (18.5 to < 25.0), overweight (25.0 to < 30.0), obesity (≥ 30.0)), Townsend deprivation index (continuous), smoking status (never-smokers, former smokers, current smokers), frequency of alcohol intake (never, occasionally, 1–2 times a week, 3–4 times a week, daily, almost daily), history of diabetes (yes or no), history of hypertension (yes or no), history of chronic obstructive pulmonary disease (yes or no), physical activity (low, moderate, high, missing) and family history of cancer (yes, no, missing).

^*c*^Number of SCLC cases in each category is listed.

^*d*^Number of LUSC cases in each category is listed.

^*e*^Number of LUAD cases in each category is listed.

We assessed the impact of GERD on lung cancer risk based on the Restricted Mean Survival Time (RMST) [23]. The results indicate that participants with GERD have a lower RMST compared to those without GERD during the 14.8-year follow-up period (all *P* <0.05) (**S5 Table**).

### 3.3 Subgroup analyses for GERD and lung cancer risk

**Fig 3:**
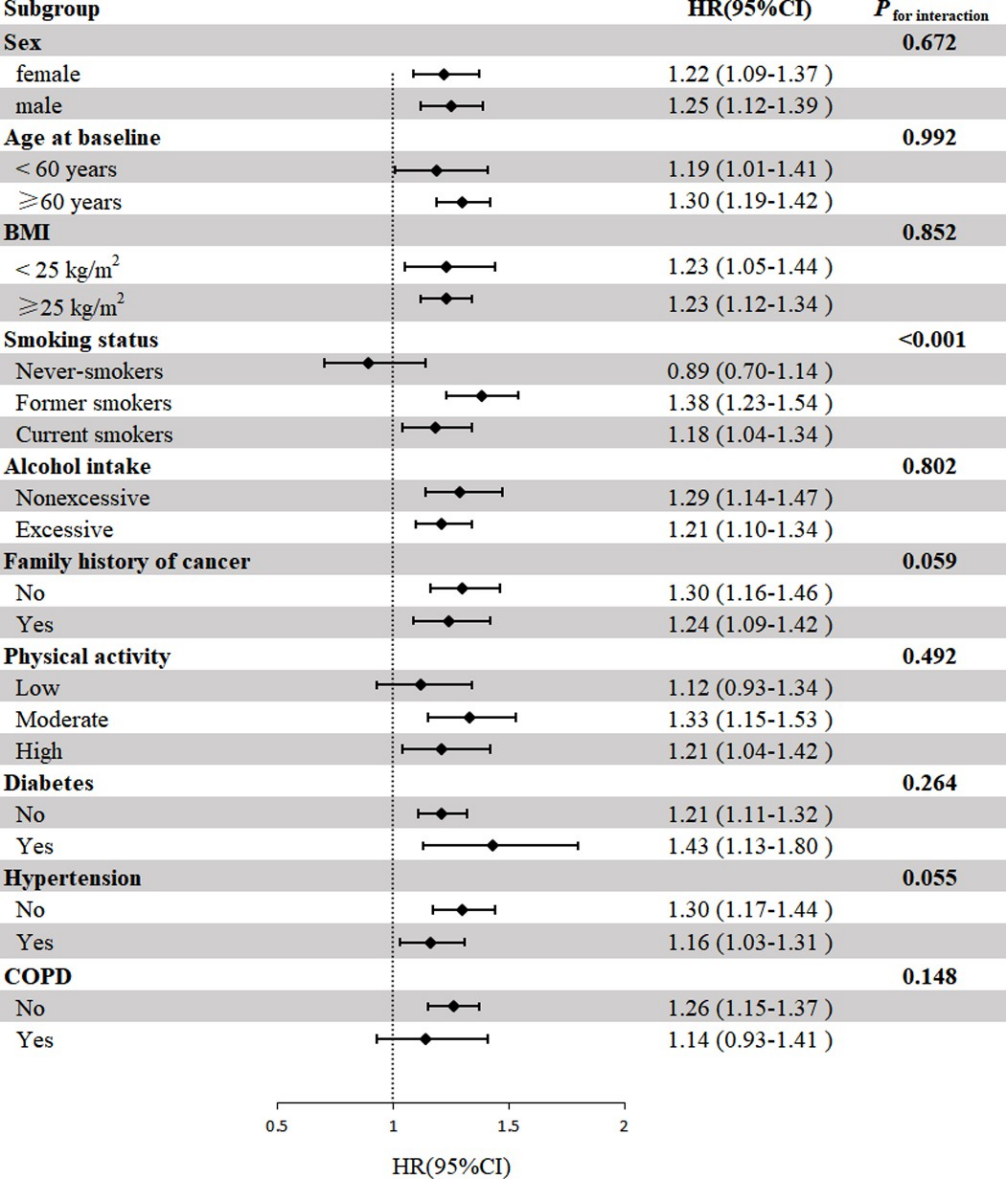
Subgroup analyses for gastroesophageal reflux disease in relation to incident lung cancer in UK Biobank. Abbreviations: HR, hazard ratio; CI, confidence interval; BMI, body mass index; COPD, chronic obstructive pulmonary disease. *Note*: The Cox proportional hazards model was used here with adjustment for the same factors as those in the model used in the main analysis. Subgrouping factors are no longer adjusted in the models. Non-excessive alcohol intake was defined as alcohol intake of less than 1–2 times a week, and excessive alcohol intake was defined as alcohol intake of at least 1–2 times a week.

We performed several predefined subgroup analyses to explore potential subgroup effects (**Fig 3**). There were statistically significant associations of GERD with lung cancer risk among former smokers (HR = 1.38, 95% CI: 1.23–1.54) and current smokers (HR = 1.18, 95% CI: 1.04–1.34), but no statistically significant association was observed among never-smokers (HR = 0.89, 95% CI: 0.70–1.14), with *P* <0.001 for the interaction term between smoking status and GERD. Among patients with GERD who had quit smoking for less than 25 years, there was an increased risk of lung cancer compared to those without GERD (HR = 1.29, 95% CI: 1.14–1.45). However, no increased risk was observed among those who had quit smoking for 25 years or more (HR = 1.18, 95% CI: 0.87–1.59) (**S6 Table**). We did not find statistically significant interactions in subgroup analyses for sex, age, BMI, alcohol intake, family history of cancer, physical activity, and the prevalence of diabetes, hypertension, and COPD (all *P* > 0.05 for interaction).

### 3.4 Accuracy analysis

The observed associations between GERD and the risk of incident lung cancer, including its various histological subtypes, remained unchanged after excluding participants diagnosed with lung cancer during the first 2 years of follow-up. Additionally, other accuracy analyses yielded consistent findings, wherein we either performed multiple imputation for missing covariates data or excluded participants with missing covariates. These accuracy analyses further confirmed the robustness of our main findings (**S7–S11 Tables**).

## 4. Discussion

In this large cross-sectional and prospective cohort study from the UK Biobank, significant positive associations were found between GERD and lung cancer, as well as risk of incident lung cancer during follow-up. Effect modification was observed among never-smokers, and the adverse effects of GERD were only observed among former and current smokers.

In the present study, we corroborated previous results on the associations between GERD and lung cancer risk. A controlled cohort study from Taiwan provided evidence that GERD may increase the risk of lung cancer [24], supporting our results. A multicenter cohort study from Northern Europe compared patients who underwent anti-reflux surgery with corresponding background population and non-operated GERD patients, respectively. The results indicated that anti-reflux operation had a reduced risk of SCLC and LUSC, but not of LUAD [11]. These two studies adjusted for smoking-related risk factors due to a lack of direct data on smoking. The positive associations we found between GERD and increased risk of lung cancer, which remained after controlling for tobacco smoking, were consistent with these two studies. A retrospective case-control study of never-smokers reported that GERD was significantly associated with non-small cell lung cancer [25]. This is not consistent with our results, as the association was observed only in smokers. MR studies have also suggested a positive association between GERD and lung cancer risk [12, 26, 27]. The causal relationship is often more complex in real-world scenarios, and our population-based study provides prospective epidemiologic evidence. More epidemiologic and clinical trial studies are needed to confirm this association in the future.

Inconsistent with mixed epidemiological results, the effect of GERD on lung cancer progression can be explained by multiple biological mechanisms. Microaspiration, a common symptom of GERD, may play a significant role in the link between these two diseases. The microaspiration of gastroesophageal reflux contents (acid, pepsin, bile acids, and pancreatic enzymes) into the airways induces chronic inflammation, which is often accompanied by the release of reactive oxygen/nitrogen species (ROS/RNS). The accumulated ROS/RNS may result in subsequent oxidative stress, potentially causing DNA damage, inhibition of apoptosis, and activation of proto-oncogenes [5, 28, 29]. Apart from malignant transformation caused by chronic inflammation, both acid and bile promote carcinogenesis by inducing DNA damage and influencing cell proliferation and apoptosis [6, 29, 30]. Consequently, it is notable that GERD may contribute to the elevated risk of lung cancer.

Our study found that the relationship was modified by smoking status. The adverse effects were observed only among former and current smokers, but not among never-smokers. Cigarette smoke induces inflammation and oxidative stress in the lung tissue, which causes lung tissue damage and a higher predisposition to lung cancer [31]. The simultaneous exposure to smoking and duodenogastric contents may exacerbate the risk of carcinogenesis in smokers. On the other side, evidence suggests that lung cancers occurring in individuals who have never smoked (LCINS) is driven by oncogenic mutations, most often involving epidermal growth factor receptor (*EGFR*) [32, 33], indicating that LCINS may be genomically and molecularly distinct from smoking-related lung cancers. The association between GERD and the risk of lung cancer with *EGFR* mutations in LCINS requires further research for confirmation. In addition, it’s worth noting that we did not find a notable association between GERD and lung cancer risk in former smokers who had quit for at least 25 years. Our findings suggested that GERD patients with a history of smoking, either former or current, may have faced a heightened risk of lung cancer, and quitting smoking remains an effective strategy for preventing lung cancer in GERD patients.

### 4.1 Comparison with other studies

GERD has been found to be significantly associated with increased risk of several respiratory diseases, including asthma, chronic cough, COPD, idiopathic pulmonary fibrosis, and pneumonia [12, 34–36], while population-based results on the associations between GERD and lung cancer are mixed. Our results, based on a large sample size and a wealth of covariates, were consistent with previous studies. Unlike previous studies, we further explored the potential variations in the association between GERD and risk of lung cancer across different subgroups, providing deeper insights into the complexity of the GERD-lung cancer link. Additionally, this study includes direct smoking data, which facilitates better control of smoking as a confounder.

### 4.2 Strengths and limitations

Compared to previous studies, strengths of this study include the use of a large cohort, which enhances the statistical power. Moreover, we adjusted for detailed demographic, lifestyle, and pre-existing condition information to control for potential confounding effects. Nevertheless, we also acknowledge several limitations. First, GERD may be asymptomatic. Definition using only diagnosis code (ICD-10/9) could lead to an overestimation of GERD. We therefore combined medication use, self-report, and clinical diagnosis to define GERD, aiming to improve the sensitivity of GERD diagnosis. However, GERD may be underestimated as patients with acid reflux issues may be receiving medication or surgical treatment. Second, the study utilized ICD-10 codes to identify cases of lung cancer, which may not effectively capture cases in their early stages and could result in underdiagnosis, potentially leading to misclassification. Third, the participants in the UK Biobank cohort are predominantly of white ethnicity, with ages concentrated between 40 and 69 years. Therefore, the findings may lack generalizability to other ethnicities or age demographics; more varied populations should be further assessed to support the generalizability of our findings. Fourth, histological subtyping could not be determined for approximately one-third of lung cancer cases due to missing data. Finally, due to the usual discrepancy between the diagnosis and treatment dates of lung cancer, there may be a time-related bias in our analysis.

## 5. Conclusions

This study aimed to investigate the association between GERD and the risk of lung cancer. We identified that GERD was associated with a greater risk of lung cancer, particularly among smokers. This finding may help to improve the intervention and treatment for both diseases.

## Supporting information

S1 Table Definitions and descriptions of exposure. (DOCX)

S2 Table Information on missing values of covariates in prospective study. (DOCX)

S3 Table Information on missing values of covariates in cross-sectional study. (DOCX)

S4 Table Baseline characteristics of participants according to the status of gastroesophageal reflux disease in the cross-sectional study. (DOCX)

S5 Table Restricted mean survival time of the study participants. (DOCX)

S6 Table Risk of incident lung cancer in relation to the gastroesophageal reflux disease according to smoking cessation duration. (DOCX)

S7 Table Sensitivity analyses estimated the risk of incident different histological subtypes of lung cancer in relation to gastroesophageal reflux disease after excluding incident lung cancer within 2 years of follow-up. (DOCX)

S8 Table Sensitivity analyses estimated the risk of incident different histological subtypes of lung cancer in relation to gastroesophageal reflux disease by multiple imputations. (DOCX)

S9 Table Sensitivity analyses estimated the risk of incident different histological subtypes of lung cancer in relation to gastroesophageal reflux disease in complete case analysis. (DOCX)

S10 Table Sensitivity analyses estimated the association between gastroesophageal reflux disease and lung cancer by multiple imputations. (DOCX)

S11 Table Sensitivity analyses estimated the association between gastroesophageal reflux disease and lung cancer in complete case analysis. (DOCX)

S1 Fig Flow diagram showing participants selection process in this study. (TIF)
