# Supplementary material for: Gastroesophageal reflux disease and risk of incident lung cancer: A large prospective cohort study in UK Biobank
Source: PLoS One. 2024 Nov 11;19(11):e0311758. doi: 10.1371/journal.pone.0311758 (PMC11554179; doi:10.1371/journal.pone.0311758)
Supplement: S1 Table — (DOCX) [file pone.0311758.s001.docx]

| **S1 Table. Definitions and descriptions of exposure** | | | |
| --- | --- | --- | --- |
| **Source** | **Description** | **Field ID** | **Code** |
| ICD10 | Diagnoses-ICD10 | 41270 | K210/K219 |
| ICD9 | Diagnoses-ICD9 | 41271 | 53010/53011 |
| OPCS | Operative procedures-OPCS4 | 41272 | G24/G25 |
| Self-report | Non-cancer illness code, self-reported | 20002 | 1138 |
| Medications, self-reported | Treatment/medication code | 20003 | 1140864752 |
|  |  |  | 1140865354 |
|  |  |  | 1140865426 |
|  |  |  | 1140865618 |
|  |  |  | 1140865634 |
|  |  |  | 1140879406 |
|  |  |  | 1140909496 |
|  |  |  | 1140909500 |
|  |  |  | 1140916980 |
|  |  |  | 1140929012 |
|  |  |  | 1141177526 |
|  |  |  | 1141177532 |
|  |  |  | 1141184376 |
