# Supplementary material for: Gastroesophageal reflux disease and risk of incident lung cancer: A large prospective cohort study in UK Biobank
Source: PLoS One. 2024 Nov 11;19(11):e0311758. doi: 10.1371/journal.pone.0311758 (PMC11554179; doi:10.1371/journal.pone.0311758)
Supplement: S2 Table — (DOCX) [file pone.0311758.s002.docx]

| **S2 Table. Information on missing values of covariates in prospective study** | | |
| --- | --- | --- |
| Characteristics | No.missing(%) | Imputation method |
| Sex | 0 | / |
| Age at recruitment | 2 (4.36×10^−4^) | Median |
| Ethnicity | 2,235 (0.49) | Mode |
| BMI | 2,074 (0.45) | Mode |
| Townsend deprivation index | 567 (0.12) | Median |
| Smoking status | 2,331 (0.51) | Mode |
| Frequency of alcohol intake | 1,066 (0.23) | Mode |
| Diabetes | 0 | / |
| Hypertension | 0 | / |
| COPD | 827 (0.18) | Mode |
| Physical activity | 90,391 (19.71) | Missing indicator |
| Family history of cancer | 54,978 (11.99) | Missing indicator |
| Abbreviations: BMI, body mass index；COPD, chronic obstructive pulmonary disease. | | |
