# Supplementary material for: Gastroesophageal reflux disease and risk of incident lung cancer: A large prospective cohort study in UK Biobank
Source: PLoS One. 2024 Nov 11;19(11):e0311758. doi: 10.1371/journal.pone.0311758 (PMC11554179; doi:10.1371/journal.pone.0311758)
Supplement: S3 Table — (DOCX) [file pone.0311758.s003.docx]

| **S3 Table. Information on missing values of covariates in cross-sectional study** | | |
| --- | --- | --- |
| Characteristics | No.missing(%) | Imputation method |
| Sex | 0 | / |
| Age at recruitment | 2 (3.99×10^−4^) | Median |
| Ethnicity | 2,421 (0.48) | Mode |
| BMI | 2,296 (0.46) | Mode |
| Townsend deprivation index | 622 (0.12) | Median |
| Smoking status | 2,602 (0.52) | Mode |
| Frequency of alcohol intake | 1,152 (0.23) | Mode |
| Diabetes | 33(6.58×10^-3^) | Mode |
| Hypertension | 33(6.58×10^-3^) | Mode |
| COPD | 925 (0.18) | Mode |
| Physical activity | 99,623 (19.86) | Missing indicator |
| Family history of cancer | 60,262 (12.01) | Missing indicator |
| Abbreviations: BMI, body mass index；COPD, chronic obstructive pulmonary disease. | | |
