# Supplementary material for: Gastroesophageal reflux disease and risk of incident lung cancer: A large prospective cohort study in UK Biobank
Source: PLoS One. 2024 Nov 11;19(11):e0311758. doi: 10.1371/journal.pone.0311758 (PMC11554179; doi:10.1371/journal.pone.0311758)
Supplement: S4 Table — (DOCX) [file pone.0311758.s004.docx]

| **S4 Table. Baseline characteristics of participants according to the status of gastroesophageal reflux disease in the cross-sectional study** | | |
| --- | --- | --- |
|  | Gastroesophageal Reflux Disease | |
| Characteristics | No | Yes |
| Total number of patients | 435,871 | 65,698 |
| Male (%) | 198,689 (45.6) | 29,942 (45.6) |
| Age at baseline (median [IQR]-year) | 57.00(50.00,63.00) | 61.00(54.00,65.00) |
| White (%) | 412,233 (94.6) | 62,441 (95.0) |
| BMI (%- kg/m^2^) |  |  |
| <18.5 | 2,388 (0.5) | 238 (0.4) |
| 18.5-<25 | 149,059 (34.2) | 13,299 (20.2) |
| 25.0-<30.0 | 185,878 (42.6) | 28,486 (43.4) |
| ≥30.0 | 98,546 (22.6) | 23,675 (36.0) |
| Townsend deprivation index (median [IQR]) | -2.18(-3.67,0.43) | -1.76(-3.44,1.32) |
| Smoking status (%) |  |  |
| Never-smokers | 244,686 (56.1) | 31,125 (47.4) |
| Former smokers | 145,919 (33.5) | 26,971 (41.1) |
| Current smokers | 45,266 (10.4) | 7,602 (11.6) |
| Frequency of alcohol intake (%) |  |  |
| Never | 32,582 (7.5) | 7,934 (12.1) |
| Occasionally*^a^* | 96,251 (22.1) | 17,477 (26.6) |
| 1-2 times a week | 114,268 (26.2) | 16,050 (24.4) |
| 3-4 times a week | 102,960 (23.6) | 12,382 (18.8) |
| Daily or almost daily | 89,810 (20.6) | 11,855 (18.0) |
| Family history of cancer (%) |  |  |
| No | 254,277 (58.3) | 35,346 (53.8) |
| Yes | 130,953 (30.0) | 20,731 (31.6) |
| Missing | 50,641 (11.6) | 9,621 (14.6) |
| Physical activity (%) |  |  |
| Low | 64,028 (14.7) | 12,076 (18.4) |
| Moderate | 144,017 (33.0) | 19,850 (30.2) |
| High | 144,003 (33.0) | 17,972 (27.4) |
| Missing | 83,823 (19.2) | 15,800 (24.0) |
| Diabetes (%) | 19,246 (4.4) | 6,229 (9.5) |
| Hypertension (%) | 107,029 (24.6) | 26,240 (39.9) |
| COPD (%) | 5,962 (1.4) | 2,849 (4.3) |
| *Notes:* Values are median (IQR) for continuous variables and frequencies (percentages) for categorical variables.  Abbreviations: BMI, body mass index; COPD, chronic obstructive pulmonary disease; IQR, inter quartile range.  *^a^* Includes individuals who drink on special occasions only and individuals who drink 1–3 times a month. | | |
