# Supplementary material for: Gastroesophageal reflux disease and risk of incident lung cancer: A large prospective cohort study in UK Biobank
Source: PLoS One. 2024 Nov 11;19(11):e0311758. doi: 10.1371/journal.pone.0311758 (PMC11554179; doi:10.1371/journal.pone.0311758)
Supplement: S5 Table — (DOCX) [file pone.0311758.s005.docx]

| **S5 Table. Restricted mean survival time (years) of the study participants** | | | | | | | |
| --- | --- | --- | --- | --- | --- | --- | --- |
|  | Lung Cancer |  | SCLC |  | LUSC |  | LUAD |
| Non-GERD | 14.734(14.732-14.737) |  | 14.794(14.793-14.79) |  | 14.788(14.787-14.789) |  | 14.773(14.771-14.774) |
| GERD | 14.671(14.662-14.680) |  | 14.787(14.784-14.78) |  | 14.768(14.764-14.773) |  | 14.752(14.745-14.758) |
| Estimate (95% CI);  P | 0.063(0.054-0.072)  <0.001 |  | 0.007(0.004-0.010)  <0.001 |  | 0.019(0.015-0.024)  <0.001 |  | 0.021(0.014-0.028)  <0.001 |
| Abbreviations: GERD, gastroesophageal reflux disease; CI, confidence interval; SCLC, small cell lung cancer; LUSC, lung squamous cell carcinoma; LUAD, lung adenocarcinoma. | | | | | | | |
