# Supplementary material for: Gastroesophageal reflux disease and risk of incident lung cancer: A large prospective cohort study in UK Biobank
Source: PLoS One. 2024 Nov 11;19(11):e0311758. doi: 10.1371/journal.pone.0311758 (PMC11554179; doi:10.1371/journal.pone.0311758)
Supplement: S6 Table — (DOCX) [file pone.0311758.s006.docx]

| **S6 Table. Risk of incident lung cancer in relation to the gastroesophageal reflux disease according to smoking cessation duration** | | |
| --- | --- | --- |
| Smoking cessation duration | HR (95% CI)*^a^* | *P* |
| <25 years | 1.29 (1.14-1.45) | <0.001 |
| ≥25 years | 1.18 (0.87-1.59) | 0.292 |
| Abbreviations: HR, hazard ratio; CI, confidence interval.  *^a^*The Cox proportional hazard models were adjusted by age (continuous), sex (male or female), race (white, non-white), body mass index (underweight (< 18.5), healthy (18.5 to < 25.0), overweight (25.0 to < 30.0), obesity (≥ 30.0)), Townsend deprivation index (continuous), frequency of alcohol intake (never, occasionally, 1-2 times a week, 3-4 times a week, daily, almost daily), history of diabetes (yes or no), history of hypertension (yes or no), history of chronic obstructive pulmonary disease (yes or no), physical activity (low, moderate, high, missing) and family history of cancer (yes, no, missing). | | |
