# Supplementary material for: Gastroesophageal reflux disease and risk of incident lung cancer: A large prospective cohort study in UK Biobank
Source: PLoS One. 2024 Nov 11;19(11):e0311758. doi: 10.1371/journal.pone.0311758 (PMC11554179; doi:10.1371/journal.pone.0311758)
Supplement: S7 Table — (DOCX) [file pone.0311758.s007.docx]

| **S7 Table. Sensitivity analyses estimated the risk of incident different histological subtypes of lung cancer in relation to gastroesophageal reflux disease after** **excluding incident lung cancer within 2 years of follow-up** | | | | | | | |
| --- | --- | --- | --- | --- | --- | --- | --- |
| GERD | Lung Cancer |  | SCLC |  | LUSC |  | LUAD |
|  | HR (95% CI)*^a^*; *P* |  | HR (95% CI)*^a^*; *P* |  | HR (95% CI)*^a^*; *P* |  | HR (95% CI)*^a^*; *P* |
| No | 1 (referent) |  | 1 (referent) |  | 1 (referent) |  | 1 (referent) |
| Yes | 1.22 (1.12-1.32); <0.001 |  | 1.40 (1.08-1.82); 0.012 |  | 1.32 (1.10-1.58); 0.003 |  | 1.16 (1.01-1.33); 0.035 |
| Abbreviations: GERD, gastroesophageal reflux disease; HR, hazard ratio; CI, confidence interval; SCLC, small cell lung cancer; LUSC, lung squamous cell carcinoma; LUAD, lung adenocarcinoma.  *^a^*The Cox proportional hazard models were adjusted by age (continuous), sex (male or female), race (white, non-white), body mass index (underweight (< 18.5), healthy (18.5 to < 25.0), overweight (25.0 to < 30.0), obesity (≥ 30.0)), Townsend deprivation index (continuous), smoking status (never-smokers, former smokers, current smokers), frequency of alcohol intake (never, occasionally, 1-2 times a week, 3-4 times a week, daily, almost daily), history of diabetes (yes or no), history of hypertension (yes or no), history of chronic obstructive pulmonary disease (yes or no), physical activity (low, moderate, high, missing) and family history of cancer (yes, no, missing). | | | | | | | |
