# Supplementary material for: Gastroesophageal reflux disease and risk of incident lung cancer: A large prospective cohort study in UK Biobank
Source: PLoS One. 2024 Nov 11;19(11):e0311758. doi: 10.1371/journal.pone.0311758 (PMC11554179; doi:10.1371/journal.pone.0311758)
Supplement: S11 Table — (DOCX) [file pone.0311758.s011.docx]

| **S11 Table. Sensitivity analyses estimated the association between gastroesophageal reflux disease and lung cancer in complete case analysis** | | | | | | | |
| --- | --- | --- | --- | --- | --- | --- | --- |
| GERD | Lung Cancer |  | SCLC |  | LUSC |  | LUAD |
|  | OR (95% CI)*^a^*; *P* |  | OR (95% CI)*^a^*; *P* |  | OR (95% CI)*^a^*; *P* |  | OR (95% CI)*^a^*; *P* |
| No | 1 (referent) |  | 1 (referent) |  | 1 (referent) |  | 1 (referent) |
| Yes | 1.87(1.34-2.58); <0.001 |  | 2.50(0.80-7.21); 0.096 |  | 2.27(1.25-4.02); 0.006 |  | 1.63(0.89-2.87); 0.103 |
| Abbreviations: GERD, gastroesophageal reflux disease; OR, odds ratio; CI, confidence interval; SCLC, small cell lung cancer; LUSC, lung squamous cell carcinoma; LUAD, lung adenocarcinoma.  *^a^*The Logistic regression models were adjusted by age (continuous), sex (male or female), race (white, non-white), body mass index (underweight (< 18.5), healthy (18.5 to < 25.0), overweight (25.0 to < 30.0), obesity (≥ 30.0)), Townsend deprivation index (continuous), smoking status (never-smokers, former smokers, current smokers), frequency of alcohol intake (never, occasionally, 1-2 times a week, 3-4 times a week, daily, almost daily), history of diabetes (yes or no), history of hypertension (yes or no), history of chronic obstructive pulmonary disease (yes or no), physical activity (low, moderate, high) and family history of cancer (yes, no). | | | | | | | |
